# Supplementary material for: Marangoni-driven deterministic formation of softer, hollow microstructures for sensitivity-enhanced tactile system
Source: Nat Commun. 2024 Jul 3;15:5596. doi: 10.1038/s41467-024-49864-z (PMC11222500; doi:10.1038/s41467-024-49864-z)
Supplement: Supplementary file 1 — Supplementary Information [file 41467_2024_49864_MOESM1_ESM.pdf]

Supplementary Information for

Marangoni-driven Deterministic Formation of Softer, Hollow Microstructures for Sensitivity-enhanced Tactile System

Wennan Xiong[1,2], Fan Zhang[1,2,*], Shiyuan Qu[1,2], Liting Yin[1,2], Kan Li[1,2], YongAn Huang[1,2,*]

[1] State Key Laboratory of Intelligent Manufacturing Equipment and Technology, Huazhong University of Science and Technology, Wuhan, 430074, P.R. China

[2] Flexible Electronics Research Center, Huazhong University of Science and Technology, Wuhan, 430074, P.R. China

Correspondence to: fanzhang@hust.edu.cn (F.Z.), yahuang@hust.edu.cn (Y.H.)

The file includes:

Supplementary Note 1. Derivation of the scaling law for the equivalent compressive stiffness.

Supplementary Note 2. Performance optimization of the hollow micro-pyramid enhanced pressure sensor.

Supplementary Note 3. Vision-assisted location of pulse-diagnosis point.

Supplementary Fig. 1. Tilt-view scanning electron microscopy (SEM) image of the hollow micro-pyramid (HMP) dielectric film.

Supplementary Fig. 2. Surface topography of the interior cavity of the micro-pyramid at five areas (center and four corners) of the microstructured array.

Supplementary Fig. 3. SEM images of hollow microstructures with different shapes.

Supplementary Fig. 4. Measurement of thickness factors of the polymer solutions with different doping ratios of $MgCl_2 \cdot 6H_2O$.

Supplementary Fig. 5. Schematic diagram of the fluorescein-tracing method used to visualize the fluid flow.

Supplementary Fig. 6. Cross-sectional SEM image array of the HMP structures with different base widths of 39 μm, 79 μm, 125 μm, 141 μm and solution volumes of 0.5 mL, 1 mL, 1.5 mL.

Supplementary Fig. 7. Measured results of hollow-to-total height ratio for the HMP structures.

Supplementary Fig. 8. Cavity profiles of the HMP structures fabricated under different base widths and volumes, where the curves and its filled area correspond to the results based on SEM images and Gaussian fitting, respectively.

Supplementary Fig. 9. Root mean square errors of Gaussian fitting for cavity profiles of the HMP structures.

Supplementary Fig. 10. Finite element analyses of the compressive deformation of micro-pyramid structures with varying height ratios of 0.3, 0.2 and 0.

Supplementary Fig. 11. Compression behavior analysis of the HMP structures by the finite element method.

Supplementary Fig. 12. Side-view SEM image of the micro-pyramid.

Supplementary Fig. 13. Schematic structures of PVA films modified by $MgCl_2 \cdot 6H_2O$.

Supplementary Fig. 14. Experimental stress-strain curves of modified PVA films with different weight ratios of $MgCl_2 \cdot 6H_2O$ to PVA.

Supplementary Fig. 15. Elastic recovery rate of the modified PVA films with different doping ratios of $MgCl_2 \cdot 6H_2O$.

Supplementary Fig. 16. Pressure response of the capacitive pressure sensor based on the HMP dielectric film.

Supplementary Fig. 17. Performance comparison among sensors applying the hollow micro pyramids and domes.

Supplementary Fig. 18. Pressure response of relative capacitance change of the hollow pyramid-based sensor with different doping ratios of $MgCl_2 \cdot 6H_2O$.

Supplementary Fig. 19. Compressive strain analysis of the hollow micro-pyramid structures under different applied pressures by the finite element method.

Supplementary Fig. 20. Relative capacitance change of the HMP-enhanced sensor with different normalized distributions, and base widths.

Supplementary Fig. 21. Sensing properties of the hollow pyramid-enhanced capacitive pressure sensor (CPS).

Supplementary Fig. 22. The response of the developed sensor under a weight of 5 g with different relative humidity and ambient temperatures.

Supplementary Fig. 23. Schematic illustration and prediction results of the vision-assisted location of pulse-diagnosis point based on a multi-output support vector algorithm.

Supplementary Fig. 24. Pressure distribution mapping of the sensing array under a cylinder, and a H-shaped acrylic plate.

Supplementary Fig. 25. Schematic illustration of the fabrication process for the capacitive pressure sensor with a HMP dielectric film.

Supplementary Fig. 26. Peel-off tests conducted to evaluate the interfacial adhesion between the HMP film and the mold.

Supplementary Fig. 27. Characterizations of pyramid molds when performing two replica-molding processes.

Supplementary Fig. 28. Schematic illustration of experimental setup for measuring pressure response of the CPS.

Supplementary Fig. 29. Uniaxial tensile test to measure materials parameters of the PVA/$MgCl_2$ film.

Supplementary Table 1. Performances comparison of CPSs fabricated with the HMP array in this work and current widely-used solid micro-pyramid array.

Supplementary Table 2. The hyperelastic parameters of the PVA/$MgCl_2$ film.

Supplementary Note 1. Derivation of the scaling law for the equivalent compressive stiffness.

The compressive behavior of the hollow micro-pyramid structure is investigated through finite element analysis (FEA) simulations to establish the relationship between equivalent compressive stiffness (k) and material (Young's modulus, E) and geometric parameters (hollow-to-total height ratio h/H, base width B, and film thickness t), denoted as $k = f(h/H, B, E, t)$. Eight-node solid elements (C3D8H) were used to model the micro-pyramid structure. The results are illustrated in Supplementary Fig. 11, where the dots represent the raw data and the solid lines are the fitted curves.

The equivalent compressive stiffness significantly decreases by 3.6 times as the height ratio increases from 0.05 to 0.6, where the fitted curve is described as $k \propto (1+h/H)\ln(h/H)$. Similarly, when the base width increases from 45 μm to 150 μm, resulting in a larger interior cavity, the equivalent compressive stiffness decreases by 1.6 times. The relationship can be effectively fitted as $k \propto 1/B$. In Supplementary Fig. 11c, a distinct linear relationship can be observed between the compressive stiffness and the Young's modulus of the dielectric film (i.e., $k \propto E$). A decrease of Young's modulus from 500 MPa to 0.5 MPa results in the linear reduction of compressive stiffness by 1000 times, which highlights the significance of doping the inorganic salt magnesium chloride ($MgCl_2$) into polyvinyl alcohol (PVA) for lower modulus. While the film thickness increases from 3 μm to 18 μm, the compressive stiffness slightly increases with a linear relationship expressed as $k \propto t + 0.5B\tan\theta$, where $B = 60$ μm and $\theta = 54.7°$. With the baseline values $E = 2$ MPa, $B = 60$ μm, $t = 8$ μm, and $h/H = 0.2$, variation of any of the parameters in $f(h/H, B, E, t)$ yields approximately the same straight line for the relationship between the equivalent compressive stiffness and four key parameters of the micro-pyramid structure.

Supplementary Note 2. Performance optimization of the hollow micro-pyramid enhanced pressure sensor.

The sensing performance of the hollow micro-pyramid (HMP) enhanced capacitive pressure sensor (CPS) is influenced by its compressibility of the dielectric layer, which is closely related to geometric and material parameters: interval space to base width (*d*/*B*), base width (*B*), hollow-to-total height ratio (*h*/*H*), and Young's modulus (*E*), as discussed in previous works [1-3]. The compressive behavior of the microstructured dielectric layer is investigated through finite element analysis (FEA) simulations, as shown in Supplementary Fig. 19.

First, from the FEA results in Supplementary Fig. 19b and c, it is observed that as the interval space (*d*) between the pyramid structures increases from *B* to 3*B*, the compressive strain proportionally increases under the same applied pressure. The experimental data in Supplementary Fig. 20a indicates that the CPS exhibits higher sensitivity when utilizing a microstructure arrayed dielectric film with a larger interval space. Therefore, increasing the interval space to a value of 2*B* or 3*B* will effectively enhance the sensitivity of the pressure sensor.

Second, Supplementary Fig. 20b demonstrates that the reduction of the pyramid size will be more favorable to obtain a higher sensitivity, and this conclusion has also been verified in other works (Supplementary Table 1). The sensor based on the micro-pyramid with a smaller base width of 39 μm exhibits a higher sensitivity. However, the interior cavity within a smaller micro-pyramid can be easily collapsed under pressure, limiting the high sensitivity to a small pressure range, as depicted in Supplementary Fig. 20b. Therefore, a hollow micro-pyramid with a base width of 79 μm was selected as the final sensor for testing in this paper.

Third, enlarging the interior cavity (e.g., increasing height ratio) of micro-pyramid structures will result in a significant increment of the compressive strain, as illustrated in Supplementary Fig. 19d. This conclusion is further corroborated by the experimental results presented in Fig. 4a of the manuscript. The height ratio is determined by two critical parameters: solution volume and curing temperature. The microstructured dielectric layer utilized for testing in Fig. 4 of the manuscript is prepared under the conditions of a solution volume of 0.5 mL and room temperature curing. Consequently, it would be beneficial to reduce the solution volume or increase the curing temperature to achieve a larger cavity.

Fourth, the compressive strain rises linearly as the modulus of modified PVA film significantly decreases (Supplementary Fig. 19e). The experiment results in Supplementary Fig. 18 present that the CPS based on the modified PVA (Young's modulus of 2.6 MPa) film with a doping ratio of 20% exhibits a higher sensitivity compared with the sensor based on the pure PVA film (Young's modulus of 839 MPa). As the doping ratio of $MgCl_2$ increases, the polymer film becomes softer, but the elastic recovery also begins to deteriorate. The modified PVA film with a doping ratio of 20%, serving as the dielectric layer of the CPS in this paper, exhibits favorable rubber-like mechanical properties, with Young's modulus of 2.6 MPa and elastic recovery rate of 99% under a tensile strain of 50% (Supplementary Fig. 15). At a doping ratio of 40%, the Young's modulus declines to 0.92 MPa with an elastic recovery of 96%. With a further increase to 60%, the elastic recovery ratio decreases to below 90% with a lower modulus of 0.63 MPa. Therefore, applying the film with a doping ratio of 40% would be a considerable strategy to achieve a higher sensitivity or a lower detection limit.

Supplementary Note 3. Vision-assisted location of pulse-diagnosis point.

It is crucial to emphasize that accurate location of the artery pulse point is vital for the autonomous pulse-diagnosis robotic system. The location data of the pulse-diagnosis point is obtained through using an image processing method. A monocular camera is mounted on the robotic hand, and each eye-in-hand capture is firstly fed to the trained hand detector module to determine the hand-knuckle points in the pixel coordinate system (PCS) [4], marked with solid green dots in Supplementary Fig. 23a. To better describe the position of the artery pulse point in the image, a hand coordinate system (HCS) is established, using the ring finger metacarpal (MCP) point as the origin. Hence, the transformation matrix ($^{\mathbf{p}}\mathbf{M}_{\mathbf{h}}$) from HCS to PCS can be expressed by

$$^{\mathbf{p}}\mathbf{M}_{\mathbf{h}} = \begin{bmatrix} \cos\theta & -\sin\theta & x^{\mathrm{p}}_{\mathrm{ring\_finger\_mcp}} \\ \sin\theta & \cos\theta & y^{\mathrm{p}}_{\mathrm{ring\_finger\_mcp}} \\ 0 & 0 & 1 \end{bmatrix}, \tag{S1}$$

and θ is the rotation angle between the coordinate systems and can be calculated by

$$\theta = -\arccos\left(\frac{x^{\mathrm{p}}_{\mathrm{pinky\_mcp}} - x^{\mathrm{p}}_{\mathrm{ring\_finger\_mcp}}}{\left\| \mathbf{S}^{\mathbf{p}}_{\mathbf{pinky\_mcp}} - \mathbf{S}^{\mathbf{p}}_{\mathbf{ring\_finger\_mcp}} \right\|} \right), \tag{S2}$$

where $\mathbf{S}^{\mathbf{p}}_{\mathbf{pinky\_mcp}}$ and $\mathbf{S}^{\mathbf{p}}_{\mathbf{ring\_finger\_mcp}}$ are the coordinates of the pinky metacarpal and ring finger metacarpal points in PCS, respectively. Subsequently, multi-output support vector regression (M-SVR), capable of outputting multiple predicted values together, is utilized to predict the position of the pulse point (**S**) in HCS [5]. Four key points, specifically the wrist, thumb carpometacarpal (CMC), index finger metacarpal, and middle finger metacarpal, serve as the inputs (**X**) of the algorithm, which can be expressed by

$$\mathbf{X} = {^{\mathbf{p}}\mathbf{M}_{\mathbf{h}}}^{-1} \left[\mathbf{S}^{\mathbf{p}}_{\mathbf{pinky\_mcp}}, \mathbf{S}^{\mathbf{p}}_{\mathbf{ring\_mcp}}, \mathbf{S}^{\mathbf{p}}_{\mathbf{middle\_mcp}}, \mathbf{S}^{\mathbf{p}}_{\mathbf{index\_mcp}} \right]. \tag{S3}$$

As a result, the pulse point is given as the output of the algorithm, which is then converted into the position in the PCS by the transformation matrix (${}^{p}\mathbf{M}_{h}$) and marked with a magenta dot in the image. Following the linear transformation to convert the pulse point into the position in the robot's base coordinate system (BCS), the computer directs the robot hand to the right position for pulse detection.

A total of 33 samples are fed to train the vision-aided location prediction model based on the M-SVR algorithm. The fitting results are presented in Supplementary Fig. 23a and b, where the coefficient of determination (R^2 score) is calculated as 0.984 (preferred to be close to 1), and the root mean square error (RMSE) and the mean absolute error (MAE) are relatively small as 10.469 pixels and 7.739 pixels, respectively. Moreover, the ratio of RMSE to MAE, equal to 1.353, is close to $\sqrt{\pi/2}$ ($\approx$ 1.253), suggesting that the fitting errors follow a normal distribution. From the training data in Supplementary Fig. 23a, the predicted pulse point (marked with a magenta circle) is closely consistent with the observed pulse point (marked with a blue circle), with specific coordinate values depicted in Supplementary Fig. 23b. These results show that the prediction model has a high goodness of fit. To further validate the prediction accuracy of this model, hand images with different gestures and/or arm sizes from an additional eight independent volunteers are processed by the trained model. The results (bottom of Supplementary Fig. 23a and Fig. 23c) indicate that the predicted pulse-diagnosis point closely accords with the observed value. The RMSE and MAE are exceptionally small and calculated as only 6.314 pixels and 3.875 pixels, respectively. These results highlight the capability of the vision-aided location prediction model to accurately predict the pulse position.

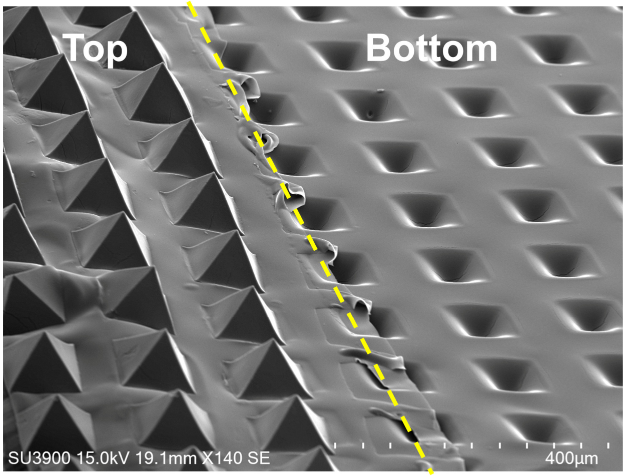

Supplementary Fig. 1. Tilt-view scanning electron microscopy (SEM) image of the hollow micro-pyramid (HMP) dielectric film.

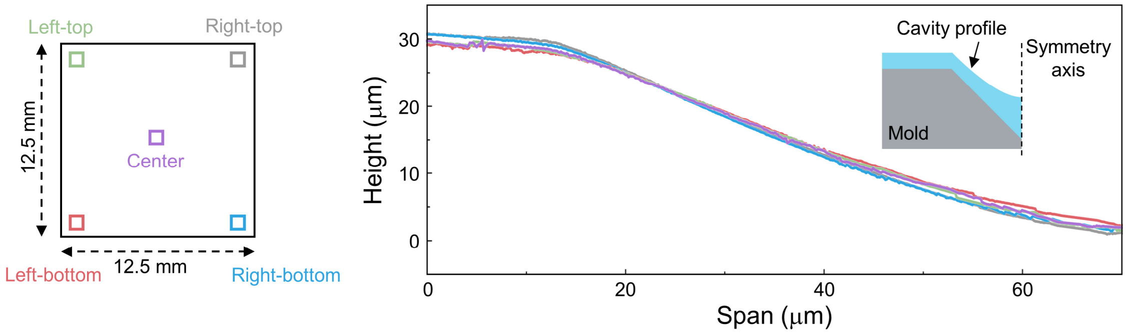

Supplementary Fig. 2. Surface topography of the interior cavity of the micro-pyramid at five areas (center and four corners) of the microstructured array.

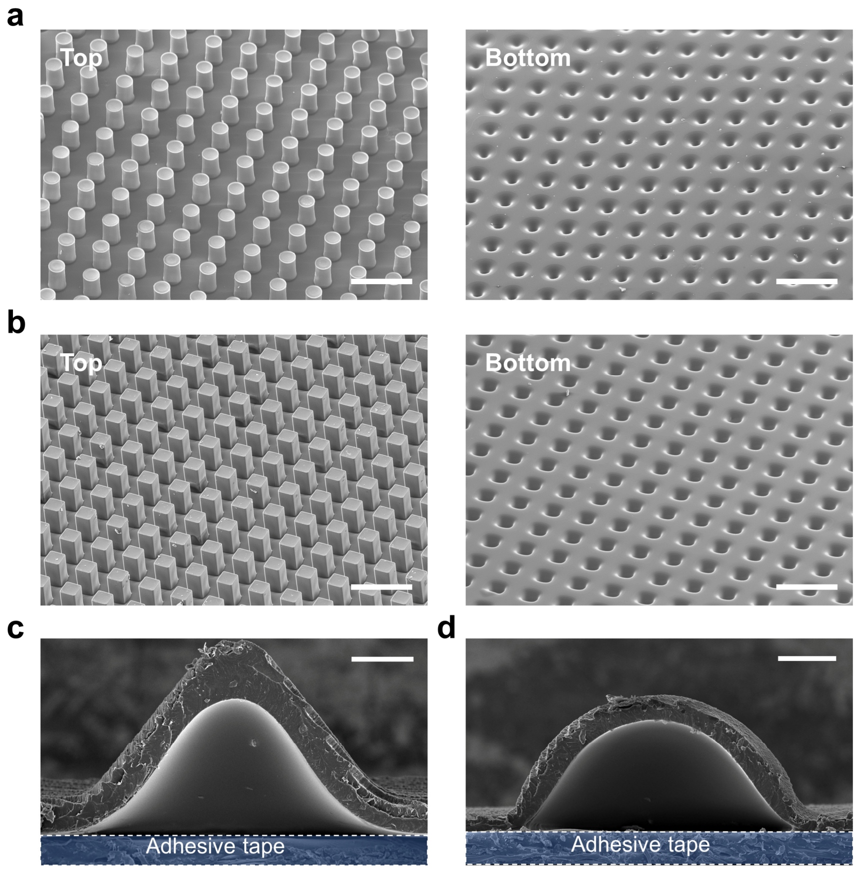

Supplementary Fig. 3. SEM images of the top and bottom surfaces of (a) the hollow micro-cylinder structure array, and **(b)** the hollow micro-cuboid structure array. **Cross-sectional SEM images of (c)** the hollow micro-pyramid structure and **(d)** the hollow micro-domo structure with a base width of 1 mm. Scale bar, 200 μm.

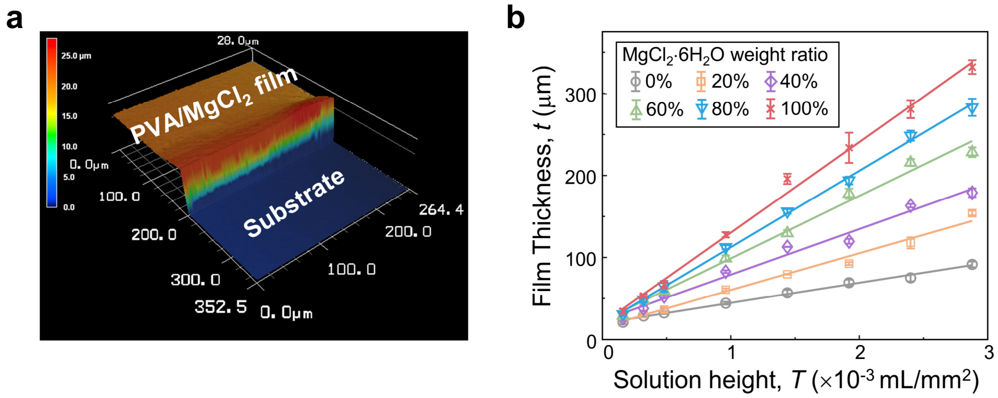

Supplementary Fig. 4. Measurement of thickness factors of the polymer solutions with different doping ratios of $MgCl_2·6H_2O$. (a) 3D laser scanning image of the polymer film curing on a glass substrate. **(b)** Linear relations between film thickness (*t*) and solution height (*T*). *T* is given by *V*/*A*, where *A* is the area of the glass substrate (25 mm × 25 mm). Error bars show s.d., $n = 3$.

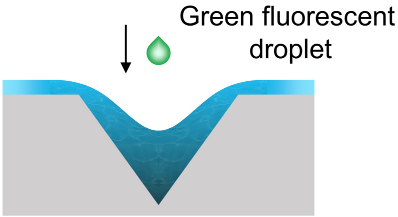

Supplementary Fig. 5. Schematic diagram of the fluorescein-tracing method used to visualize the fluid flow. A diluted green fluorescent droplet is added to the polymer solution as it evaporates to half the height of the inverted pyramid.

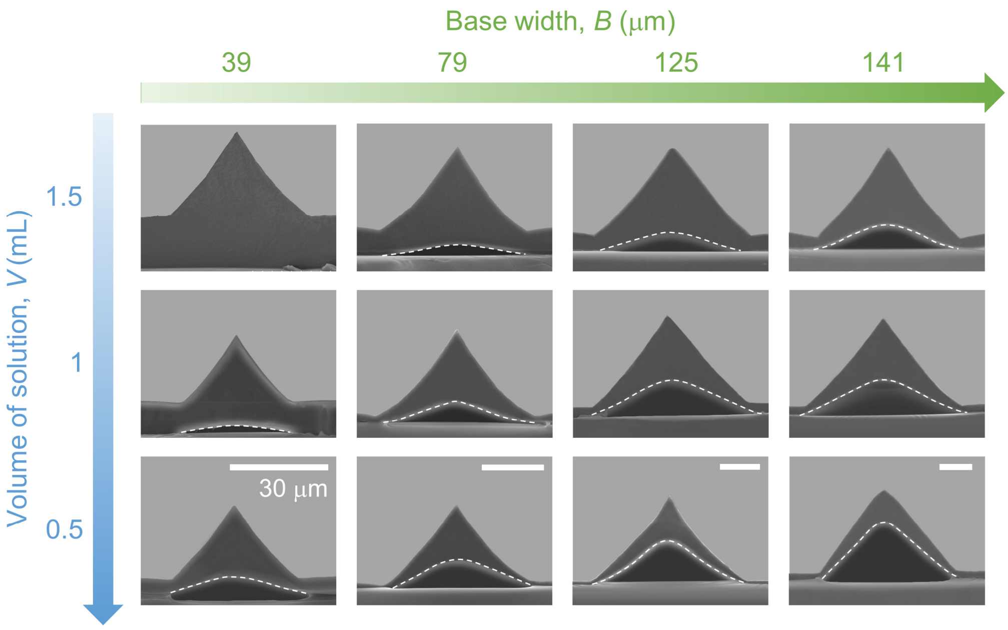

Supplementary Fig. 6. Cross-sectional SEM image array of the HMP structures with different base widths (*B*) of 39 μm, 79 μm, 125 μm, 141 μm and solution volumes (*V*) of 0.5 mL, 1 mL, 1.5 mL.

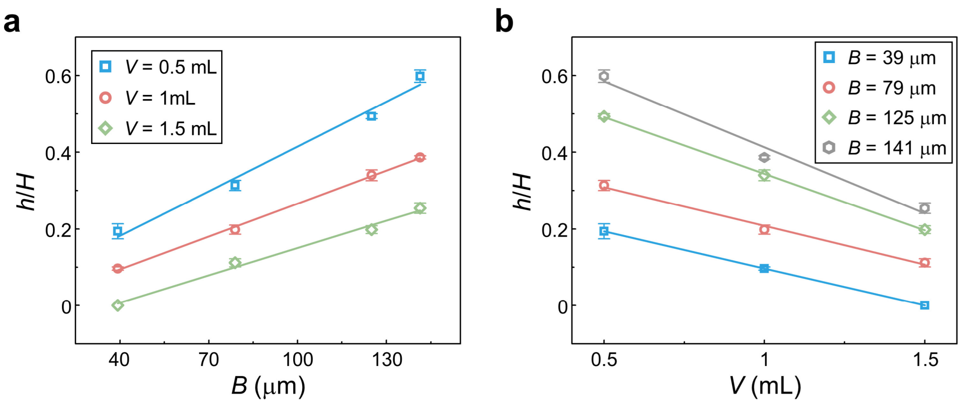

Supplementary Fig. 7. Measured results of hollow-to-total height ratio (*h*/*H*) for the HMP structures. It shows linear relationships with **(a)** the base width (*B*) of the micro-pyramid, and **(b)** the solution volume (*V*), respectively. Error bars show s.d., $n = 3$.

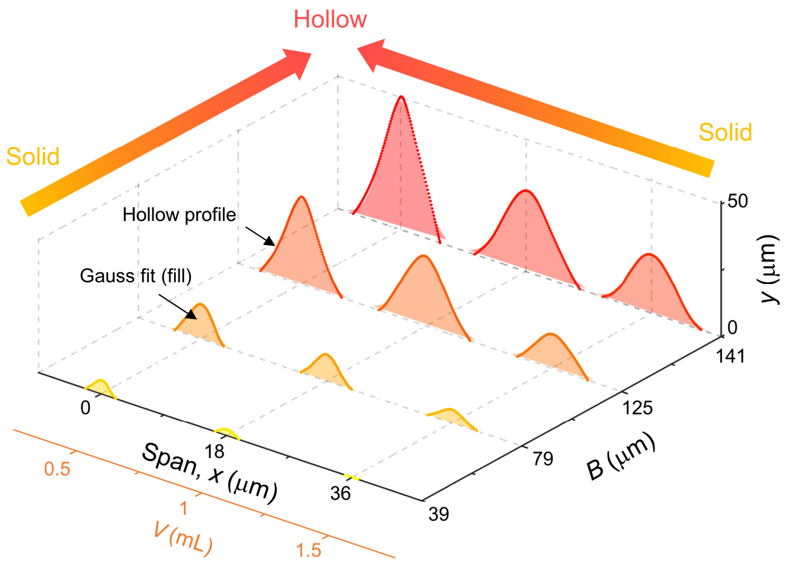

Supplementary Fig. 8. Cavity profiles of the HMP structures fabricated under different base widths and volumes, where the curves and its filled area correspond to the results based on SEM images and Gaussian fitting, respectively.

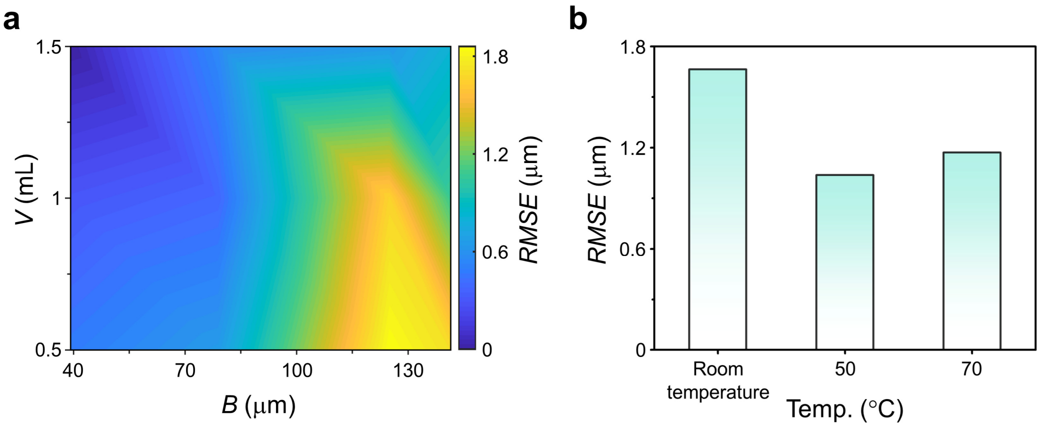

Supplementary Fig. 9. Root mean square errors (RMSEs) of Gaussian fitting for cavity profiles of the HMP structures. (a) Contour plot of RMSEs in terms of base width and solution volume. **(b)** RMSEs for the HMP structures under different curing temperatures.

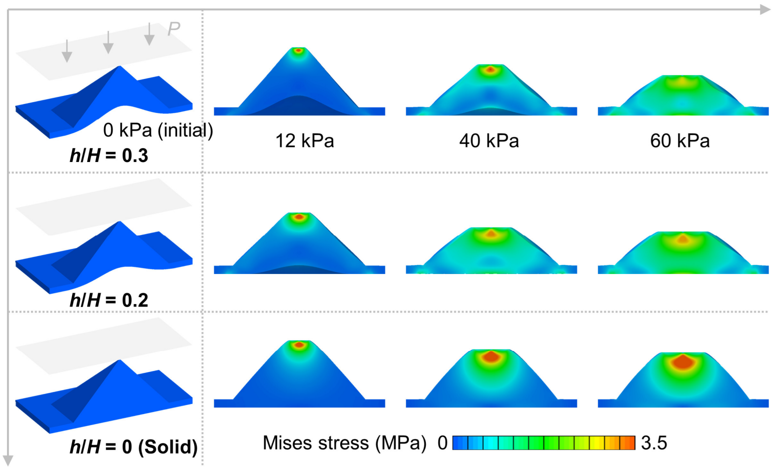

Supplementary Fig. 10. Finite element analyses of the compressive deformation of micro-pyramid structures with varying height ratios of 0.3, 0.2 and 0.

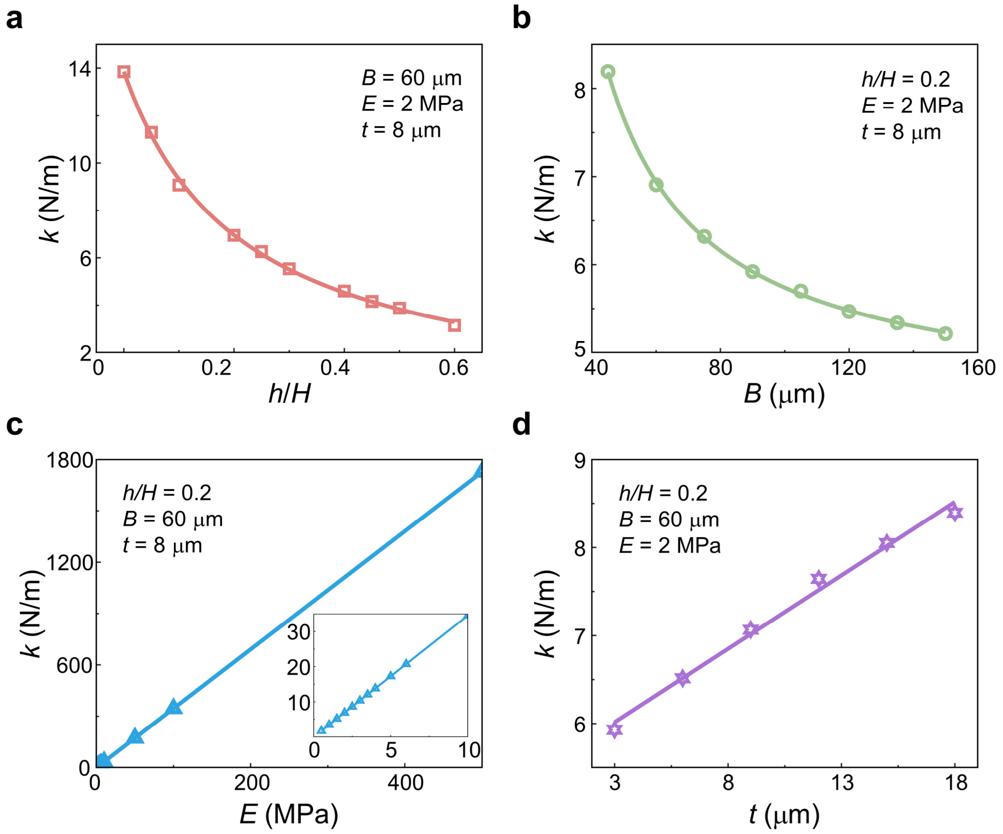

Supplementary Fig. 11. Compression behavior analysis of the HMP structures by the finite element method. Relationships between the equivalent compressive stiffness (k) of the HMP structure and its geometric and material parameters of **(a)** hollow-to-total height ratio (h/H), **(b)** base width (B), **(c)** Young's modulus (E), and **(d)** film thickness (t). The dots represent the raw data, and the solid lines are the fitted curves. The fitted curves can be described as $k \propto (1+h/H)\ln(h/H)$, $1/B$, E, and t.

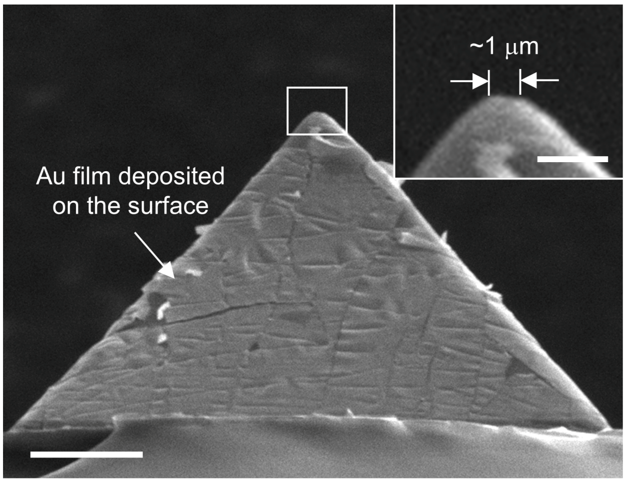

Supplementary Fig. 12. Side-view SEM image of the micro-pyramid. Scale bar, 10 μm. The inset shows the zoom-in micro-pyramidal tip structure. Scale bar, 2 μm.

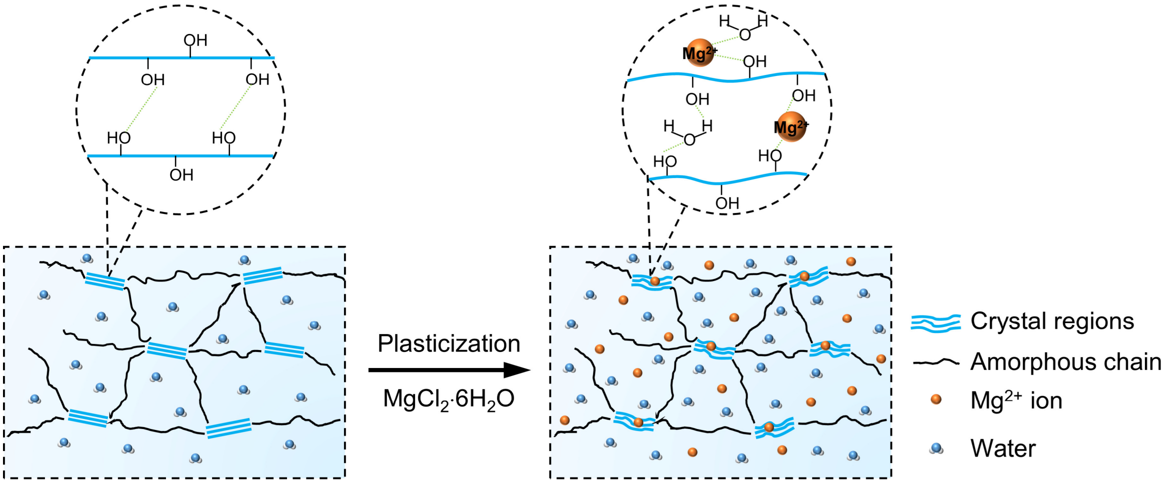

Supplementary Fig. 13. Schematic structures of PVA films modified by $MgCl_2 \cdot 6H_2O$.

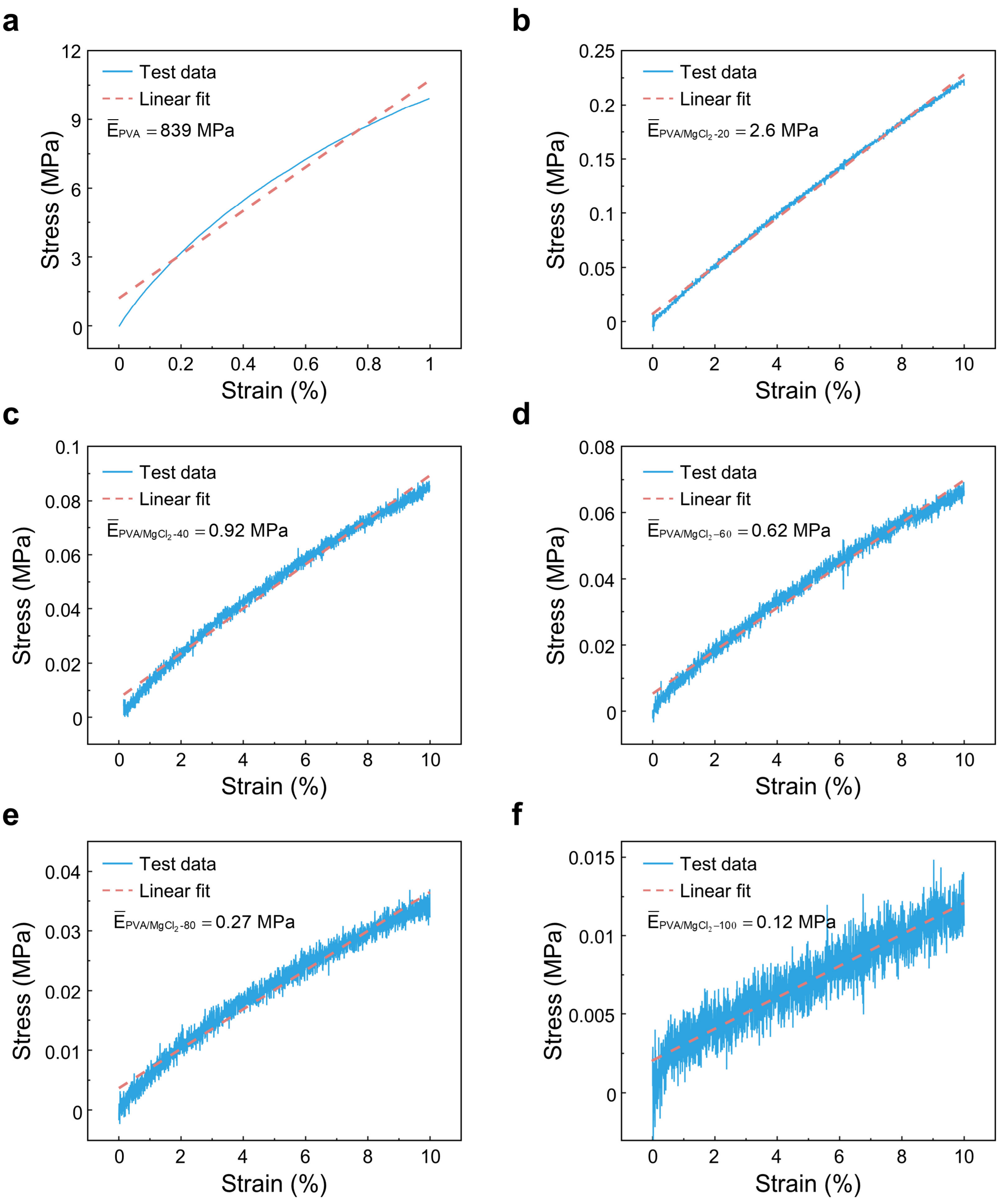

Supplementary Fig. 14. Experimental stress-strain curves of modified PVA films with different weight ratios of $MgCl_2{\cdot}6H_2O$ to PVA. (a) 0%, **(b)** 20%, **(c)** 40%, **(d)** 60%, **(e)** 80%, **(f)** 100%.

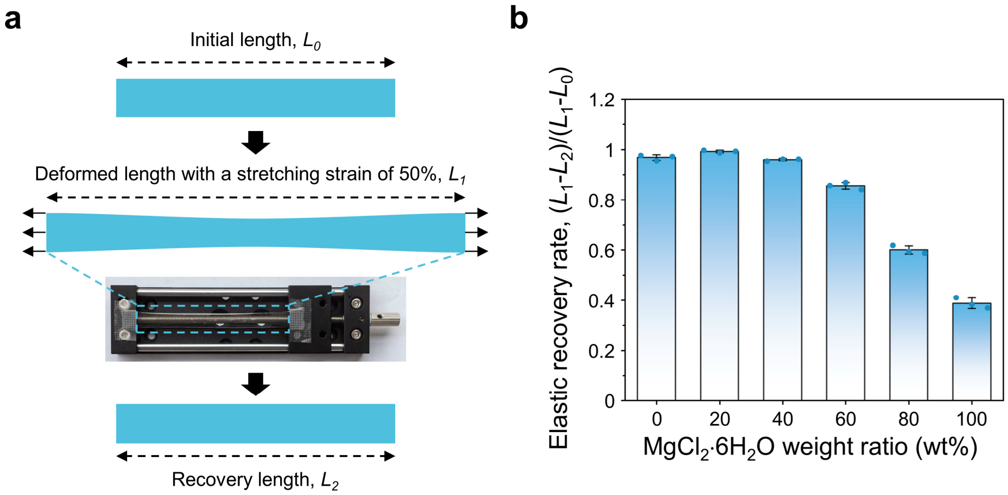

Supplementary Fig. 15. Elastic recovery rate of the modified PVA films with different doping ratios of $MgCl_2 \cdot 6H_2O$. **(a)** Schematic illustration and parameters (initial length L_0, deformed length L_1 with a stretching strain of 50%, and recovery length L_2) of elastic recovery rate experiments. **(b)** Elastic recovery rate of the modified PVA film under different levels of $MgCl_2 \cdot 6H_2O$ weight ratio. Error bars show s.d., $n = 3$.

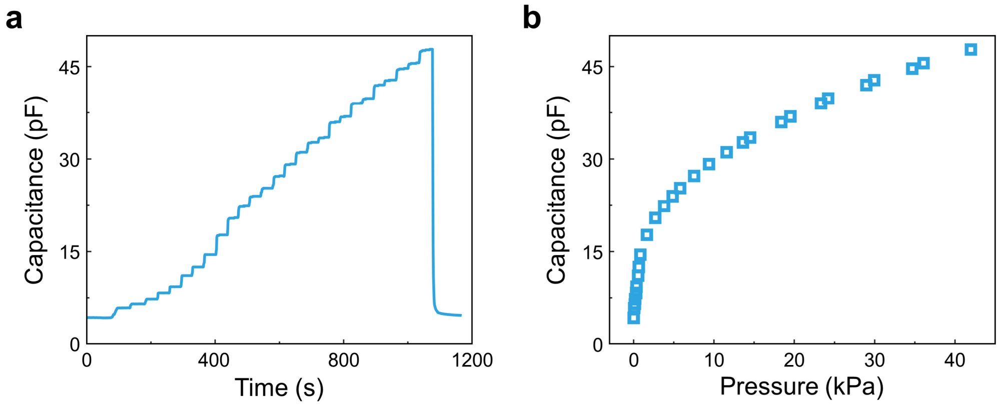

Supplementary Fig. 16. Pressure response of the capacitive pressure sensor based on the HMP dielectric film. The hollow-to-total height ratio of the micro-pyramid is 0.31 and the base width is 79 μm. **(a)** Real-time absolute capacitance under step-increasing pressures. **(b)** Averaged capacitance values versus applied pressures.

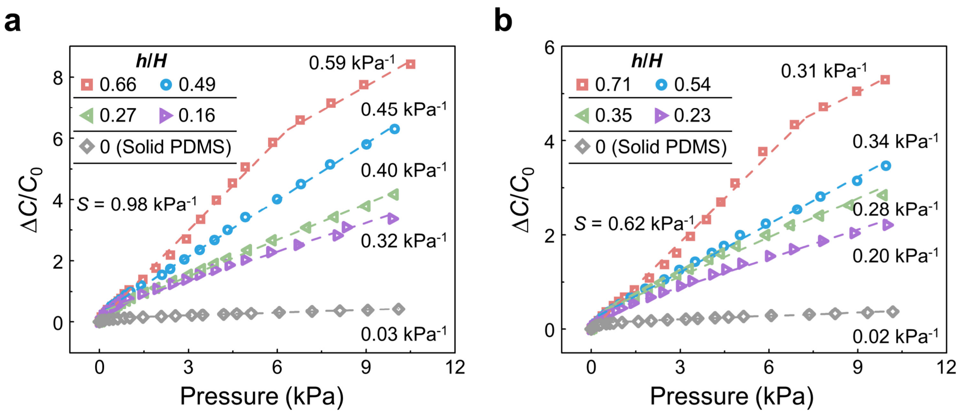

Supplementary Fig. 17. Performance comparison among sensors applying the hollow micro pyramids and domes. Relative capacitance variation with the pressure among (a) pyramid-based (Supplementary Fig. 3c) and **(b)** dome-based (Supplementary Fig. 3d) sensors with different hollow-to-total height ratios. The solid PDMS film ($h/H = 0$) has a thickness of around 200 μm, which is comparable to the thickness of the HMP structure ($h/H = 0.16$), as well as the micro-dome structure ($h/H = 0.23$).

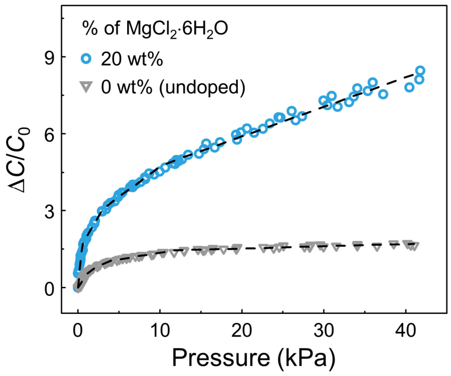

Supplementary Fig. 18. Pressure response of relative capacitance change of the hollow pyramid-based sensor with different doping ratios of $MgCl_2·6H_2O$. The pressure sensor, with a 20 wt% doping ratio of $MgCl_2·6H_2O$, exhibits sensitivities of 3.27 kPa^{-1} within 0–0.5 kPa, 0.55 kPa^{-1} within 0.5–3 kPa, 0.24 kPa^{-1} within 3–10 kPa, and 0.11 kPa^{-1} within 10–40 kPa. In comparison, the undoped pressure sensor demonstrates sensitivities of 0.48 kPa^{-1} within 0–1.3 kPa, 0.17 kPa^{-1} within 1.3–3.6 kPa, 0.052 kPa^{-1} within 3.6–11.7 kPa, and 0.0088 kPa^{-1} within 11.7–40 kPa.

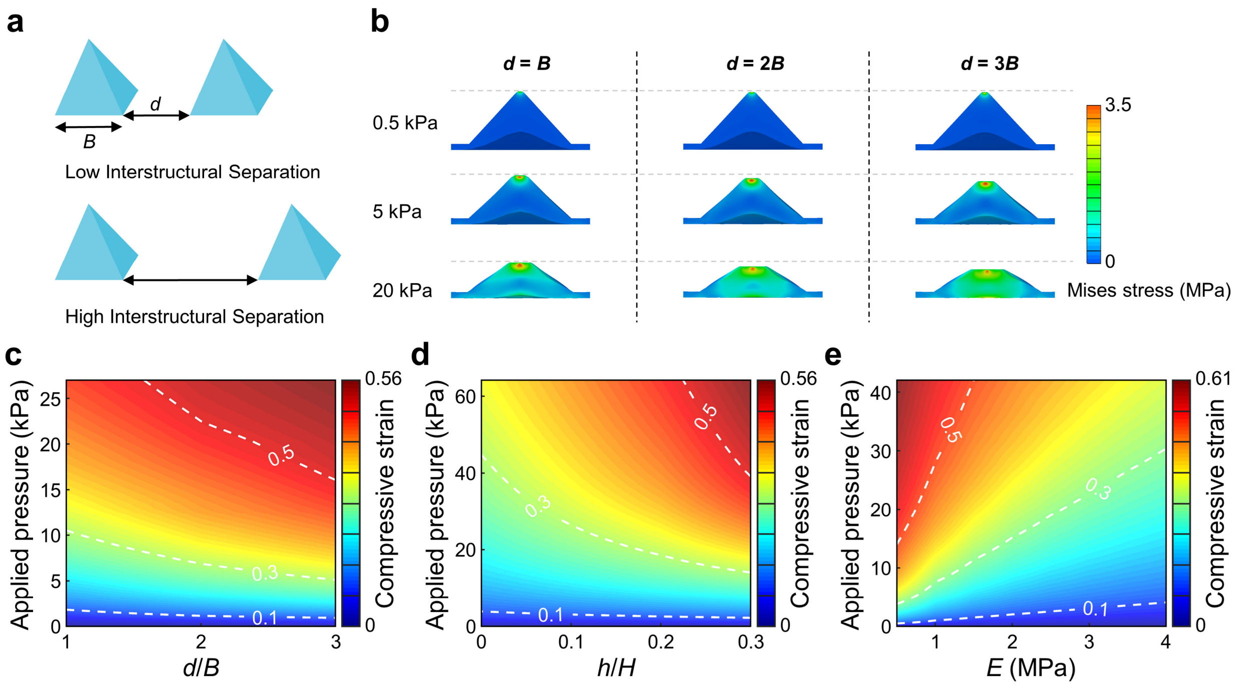

Supplementary Fig. 19. Compressive strain analysis of the hollow micro-pyramid structures under different applied pressures by the finite element method. (a) Schematic illustration of the distribution of hollow microstructures, including base width (B) and interval space (d). **(b)** FEA results of compressive deformation of the micro-pyramid structures with various interval spaces under different levels of applied pressure (0.5 kPa, 5 kPa, and 20 kPa). **(c)** Contour plot of the compressive strain of the micro-pyramid structure in terms of applied pressure and d/B for $h/H = 0.3$ and $E = 2.6$ MPa. **(d)** Contour plot of the compressive strain of the micro-pyramid structure in terms of applied pressure and h/H for $d/B = 1$ and $E = 2.6$ MPa. **(e)** Contour plot of the compressive strain of the micro-pyramid structure in terms of applied pressure and E for $h/H = 0.2$ and $d/B = 1$.

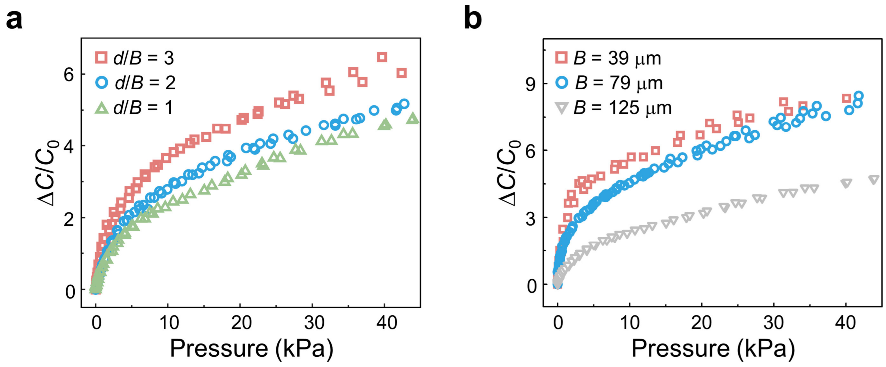

Supplementary Fig. 20. Relative capacitance change of the HMP-enhanced sensor with different (a) normalized distributions (d/B), and **(b)** base widths (B).

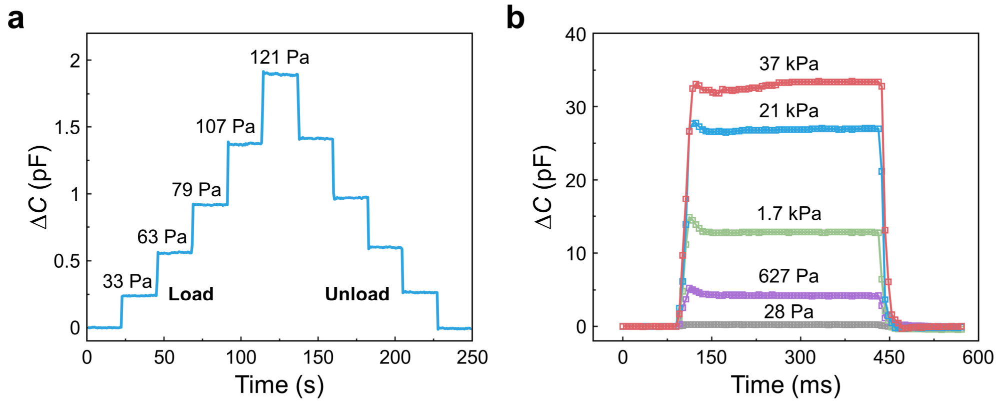

Supplementary Fig. 21. Sensing properties of the hollow pyramid-enhanced CPS. (a) Static response under stepped loading−unloading pressures from 33 Pa to 121 Pa. **(b)** Response and recovery tests under various applied pressures from a small pressure (28 Pa) to a large pressure (37 kPa).

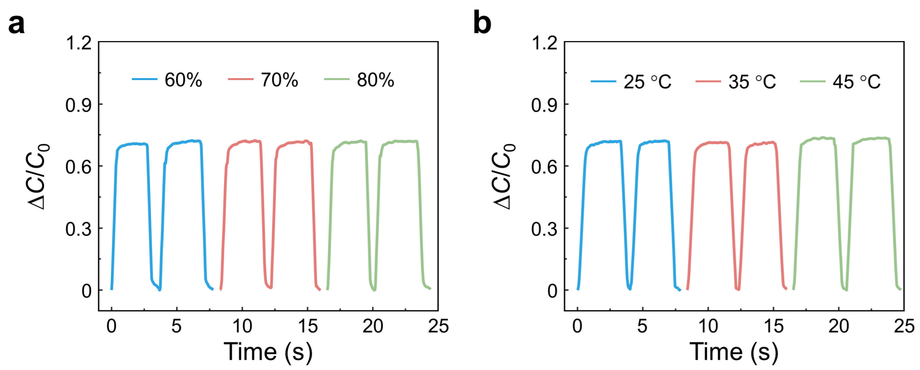

Supplementary Fig. 22. The response of the developed sensor under a weight of 5 g with different (a) relative humidity (60%, 70% and 80%), and **(b)** ambient temperatures (25 °C, 35 °C, and 45 °C).

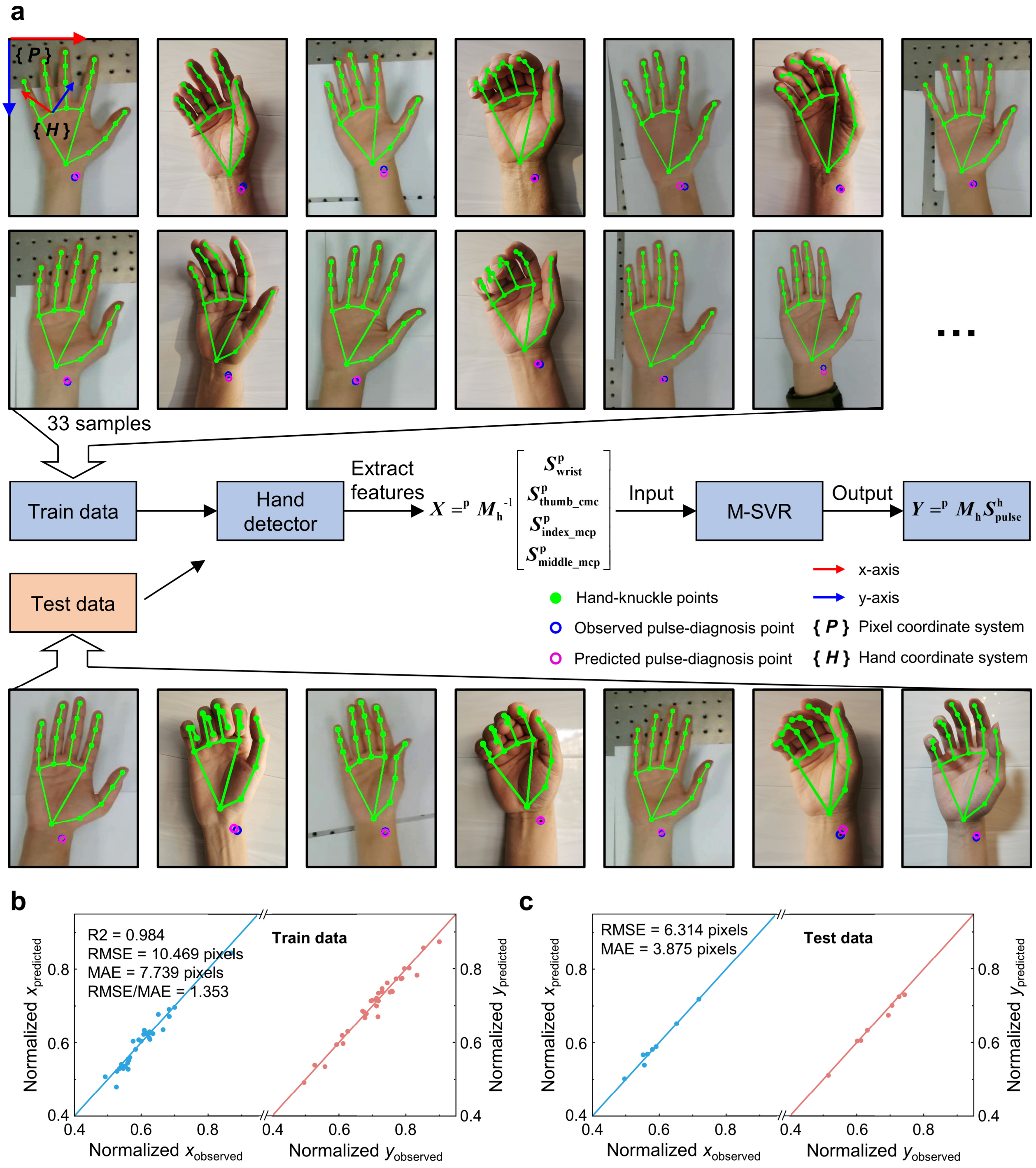

Supplementary Fig. 23. Schematic illustration and prediction results of the vision-assisted location of pulse-diagnosis point based on a multi-output support vector (M-SVR) algorithm. (a) The schematic workflow of the vision-aided location model. **(b)** Training results of the M-SVR model with 33 sets of data points, evaluated by the parameters of R^2 score, root mean square error (RMSE), mean absolute error (MAE) and RSME/MAE. **(c)** Prediction results of the M-SVR model with test data from additional eight independent volunteers, showing a high prediction accuracy.

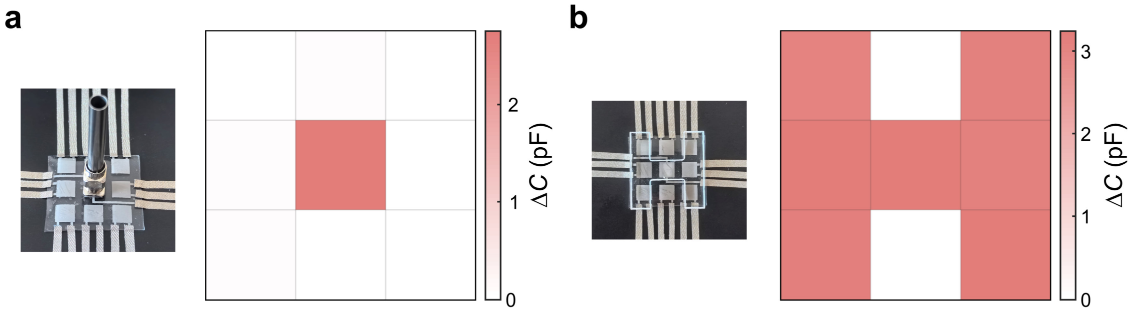

Supplementary Fig. 24. Pressure distribution mapping of the sensing array under (a) a cylinder, and **(b)** a H-shaped acrylic plate.

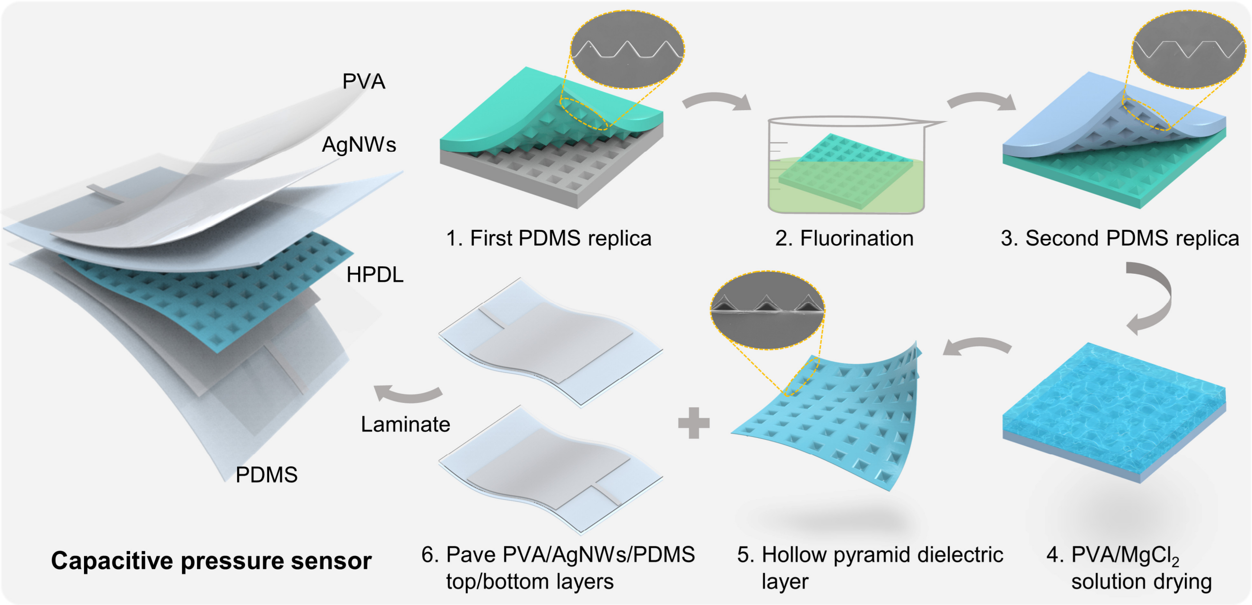

Supplementary Fig. 25. Schematic illustration of the fabrication process for the capacitive pressure sensor with a HMP dielectric film.

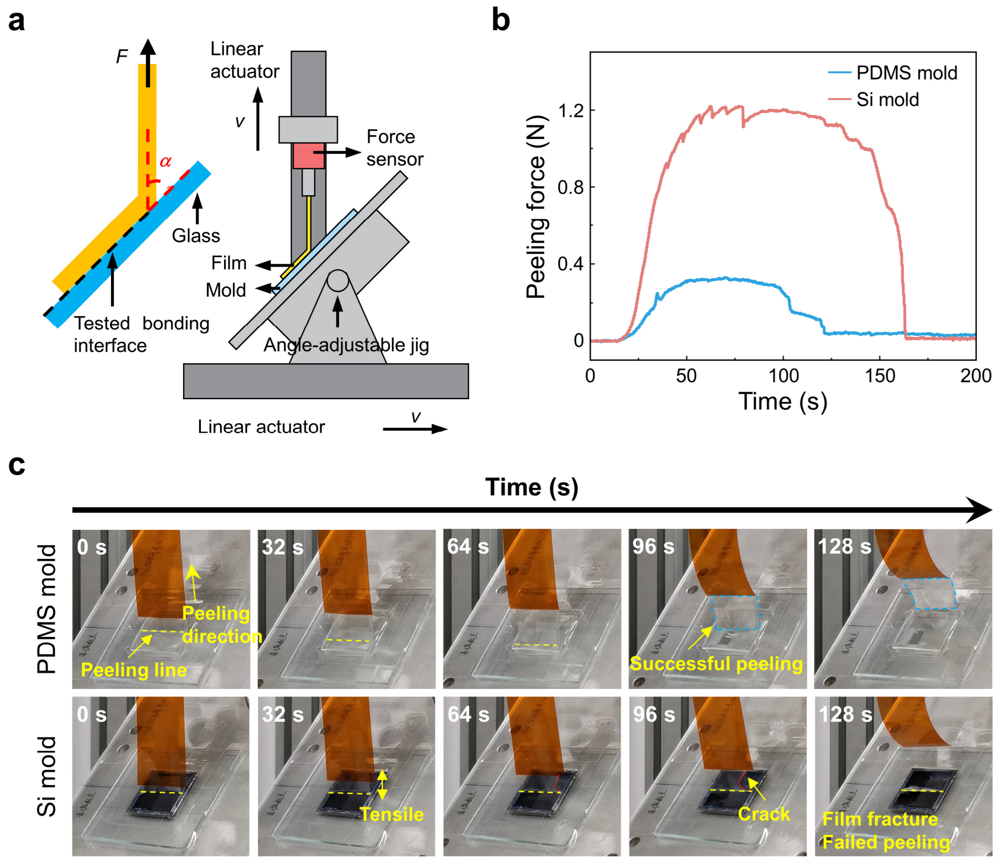

Supplementary Fig. 26. Peel-off tests conducted to evaluate the interfacial adhesion between the HMP film and the mold. (a) Schematic diagram of the self-developed peel test platform. **(b)** Comparison of peeling force when using the Si mold with a prior fluorination treatment and the PDMS mold. **(c)** Optical photographs of the peel procedures.

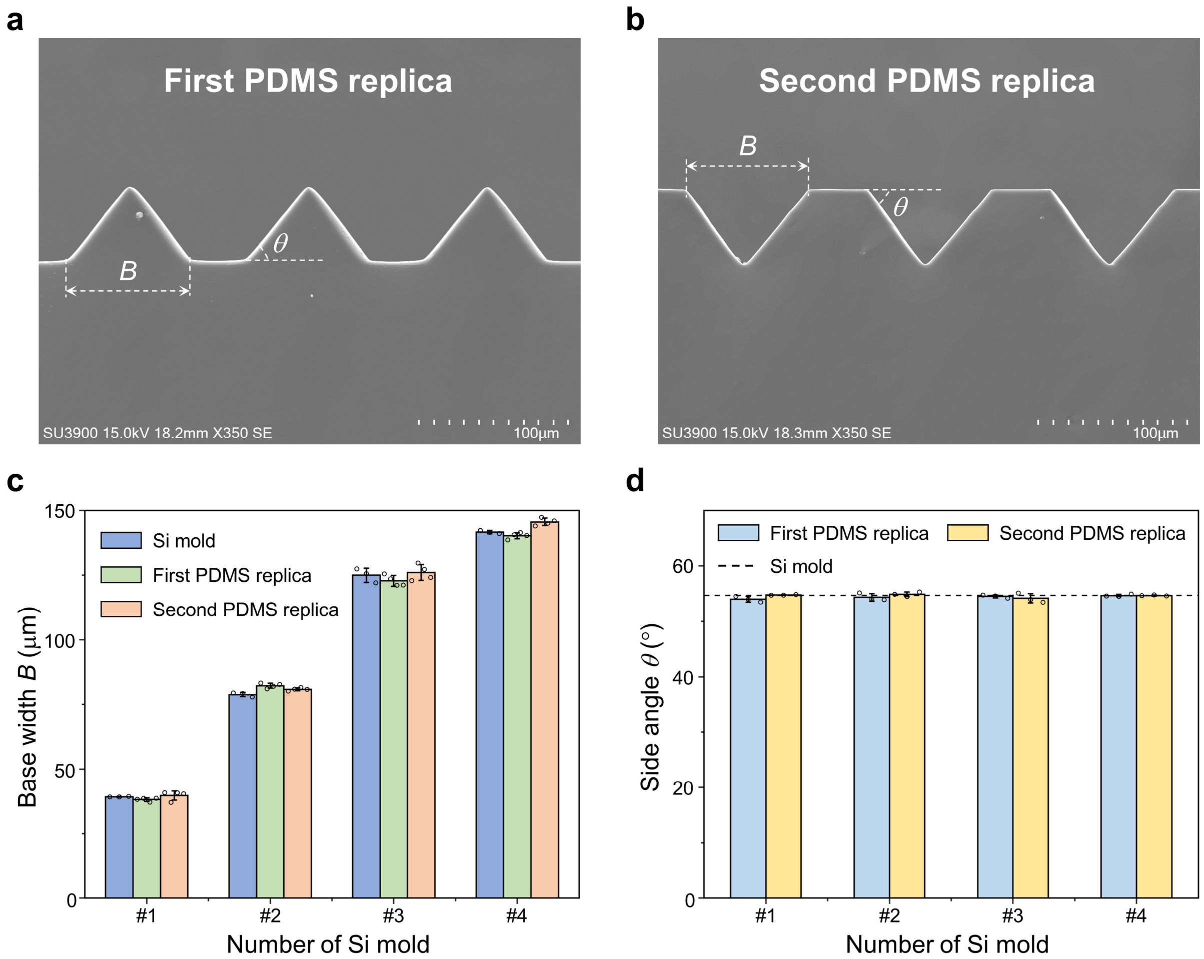

Supplementary Fig. 27. Characterizations of pyramid molds when performing two replica-molding processes. Cross-sectional SEM images of **(a)** the first PDMS mold, and **(b)** the second PDMS mold. Comparisons of **(c)** base widths and **(d)** side angles of the Si molds, first and second PDMS molds. The numbers from #1 to #4 represent the Si molds with different base widths of 39 μm, 79 μm, 125 μm, 141 μm, respectively. The side angle of Si pyramid mold is 54.7° due to the different etch rates along the silicon crystallographic planes. Error bars show s.d., $n = 3$.

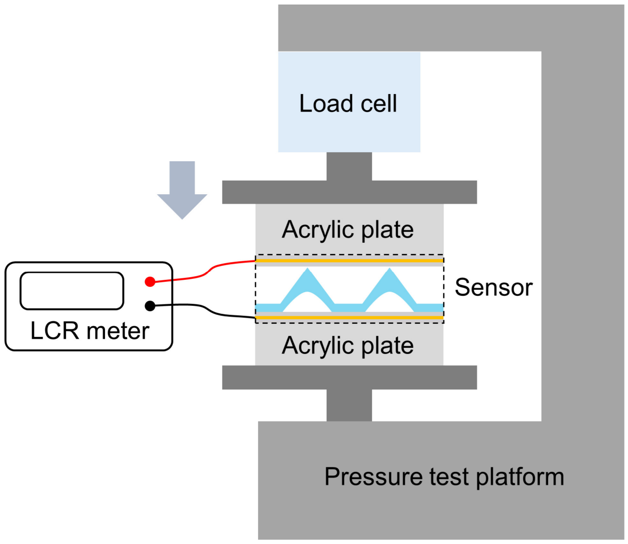

Supplementary Fig. 28. Schematic illustration of experimental setup for measuring pressure response of the CPS.

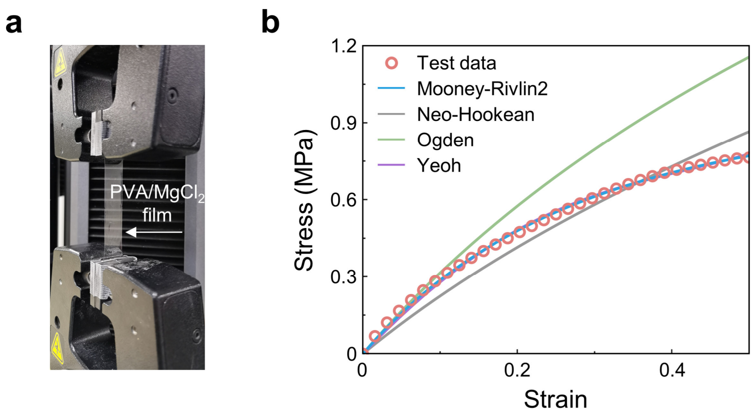

Supplementary Fig. 29. Uniaxial tensile test to measure materials parameters of the PVA/$MgCl_2$ film. (a) Photograph of the standard uniaxial tensile test. **(b)** Stress-strain curve of the PVA/$MgCl_2$ film with a weight ratio of $MgCl_2 \cdot 6H_2O$ to PVA of 20%. The test data is curve-fitted by several hyperelastic material models, and Mooney-Rivlin2 has the best fitting performances. The obtained material parameters are shown in Supplementary Table 2.

Supplementary Table 1. Performances comparison of CPSs fabricated with the HMP array in this work and current widely-used solid micro-pyramid array.

Height (μm)	Base width (μm)	Spacing（μm）	Sensitivity (kPa^{-1}) (pressure range kPa)	Reference
2	4	5.6	0.7 (< 1)	[6]
7.6	11.5	–	0.831 (<0.5)	[7]
11	15	5/10/30	0.4/0.43/0.7 (< 1)	[8]
34	55	41/88/182	0.125/0.16/0.2 (< 4)	[9]
420	600	1000	0.19 (< 1.2)	[10]
**56**	**79**	**79**	**3.45 (< 0.6)**<br>**1.11 (0.6~2)**	**This work**
**706**	**1000**	**1000**	**0.98 (< 6)**<br>**0.59 (6~10)**	

Supplementary Table 2. The hyperelastic parameters of the PVA/$MgCl_2$ film.

Mooney-Rivlin 2		
C_{10} (Pa)	C_{01} (Pa)	D_1 (Pa^{-1})
-70734	653489	0

Supplementary References

1. Wan, Y., *et al.* A Highly Sensitive Flexible Capacitive Tactile Sensor with Sparse and High-Aspect-Ratio Microstructures. *Adv. Electron. Mater.* **4**, 1700586 (2018).

2. Ruth, S. R. A., *et al.* Rational design of capacitive pressure sensors based on pyramidal microstructures for specialized monitoring of biosignals. *Adv. Funct. Mater.* **30**, 1903100 (2020).

3. Ruth, S. R. A. & Bao, Z. Designing tunable capacitive pressure sensors based on material properties and microstructure geometry. *ACS Appl. Mater. Interfaces* **12**, 58301-58316 (2020).

4. Zhang, F., *et al.* Mediapipe hands: On-device real-time hand tracking. *arXiv preprint arXiv:2006.10214*, (2020).

5. Sánchez-Fernández, M., de-Prado-Cumplido, M., Arenas-García, J. & Pérez-Cruz, F. SVM multiregression for nonlinear channel estimation in multiple-input multiple-output systems. *IEEE Trans. Signal Process.* **52**, 2298-2307 (2004).

6. Boutry, C. M., *et al.* A stretchable and biodegradable strain and pressure sensor for orthopaedic application. *Nat. Electron.* **1**, 314-321 (2018).

7. Shi, R., Lou, Z., Chen, S. & Shen, G. Flexible and transparent capacitive pressure sensor with patterned microstructured composite rubber dielectric for wearable touch keyboard application. *Sci. China Mater.* **61**, 1587-1595 (2018).

8. Luo, S., *et al.* Tunable-sensitivity flexible pressure sensor based on graphene transparent electrode. *Solid·State Electron.* **145**, 29-33 (2018).

9. Tee, B. C. K., *et al.* Tunable flexible pressure sensors using microstructured elastomer geometries for intuitive electronics. *Adv. Funct. Mater.* **24**, 5427-5434 (2014).

10. Li, Z., *et al.* Gelatin Methacryloyl‐Based Tactile Sensors for Medical Wearables. *Adv. Funct.*

Mater. **30**, 2003601 (2020).
