# Supplementary material for: Marangoni-driven deterministic formation of softer, hollow microstructures for sensitivity-enhanced tactile system
Source: Nat Commun. 2024 Jul 3;15:5596. doi: 10.1038/s41467-024-49864-z (PMC11222500; doi:10.1038/s41467-024-49864-z)
Supplement: Supplementary file 3 — Description of Additional Supplementary Files [file 41467_2024_49864_MOESM3_ESM.pdf]

Description of Additional Supplementary Files

File Name: Supplementary Movie 1
Description: Demonstration of Pulse-diagnosis robot
