# Supplementary material for: Marangoni-driven deterministic formation of softer, hollow microstructures for sensitivity-enhanced tactile system
Source: Nat Commun. 2024 Jul 3;15:5596. doi: 10.1038/s41467-024-49864-z (PMC11222500; doi:10.1038/s41467-024-49864-z)
Supplement: Supplementary file 5 — Reporting Summary [file 41467_2024_49864_MOESM5_ESM.pdf]

nature portfolio

Corresponding author(s): YongAn Huang

Last updated by author(s): 2024-05-26

Reporting Summary

Nature Portfolio wishes to improve the reproducibility of the work that we publish. This form provides structure for consistency and transparency in reporting. For further information on Nature Portfolio policies, see our Editorial Policies and the Editorial Policy Checklist.

Statistics

For all statistical analyses, confirm that the following items are present in the figure legend, table legend, main text, or Methods section.

n/a	Confirmed	
☐	☒	The exact sample size (n) for each experimental group/condition, given as a discrete number and unit of measurement
☐	☒	A statement on whether measurements were taken from distinct samples or whether the same sample was measured repeatedly
☒	☐	The statistical test(s) used AND whether they are one- or two-sided<br>*Only common tests should be described solely by name; describe more complex techniques in the Methods section.*
☒	☐	A description of all covariates tested
☒	☐	A description of any assumptions or corrections, such as tests of normality and adjustment for multiple comparisons
☒	☐	A full description of the statistical parameters including central tendency (e.g. means) or other basic estimates (e.g. regression coefficient) AND variation (e.g. standard deviation) or associated estimates of uncertainty (e.g. confidence intervals)
☒	☐	For null hypothesis testing, the test statistic (e.g. F, t, r) with confidence intervals, effect sizes, degrees of freedom and P value noted<br>*Give P values as exact values whenever suitable.*
☒	☐	For Bayesian analysis, information on the choice of priors and Markov chain Monte Carlo settings
☒	☐	For hierarchical and complex designs, identification of the appropriate level for tests and full reporting of outcomes
☒	☐	Estimates of effect sizes (e.g. Cohen's d, Pearson's r), indicating how they were calculated

Our web collection on statistics for biologists contains articles on many of the points above.

Software and code

Policy information about availability of computer code

Data collection	LabVIEW 2017 was used to collect experimental data.
Data analysis	Origin9_64 and Microsoft Powerpoint 16 were used to plot the figure of manuscript.<br>ABAQUS 6.14-1 was used for finite element analysis simulation.

For manuscripts utilizing custom algorithms or software that are central to the research but not yet described in published literature, software must be made available to editors and reviewers. We strongly encourage code deposition in a community repository (e.g. GitHub). See the Nature Portfolio guidelines for submitting code & software for further information.

Data

Policy information about availability of data

All manuscripts must include a data availability statement. This statement should provide the following information, where applicable:

- Accession codes, unique identifiers, or web links for publicly available datasets
- A description of any restrictions on data availability
- For clinical datasets or third party data, please ensure that the statement adheres to our policy

The data that support the findings of this study are available within this article and its Supplementary Information, and from the corresponding authors upon reasonable request.

Research involving human participants, their data, or biological material

Policy information about studies with human participants or human data. See also policy information about sex, gender (identity/presentation), and sexual orientation and race, ethnicity and racism.

Reporting on sex and gender	The study (Figure 5) serves as a proof of concept for robot tactile sensing and demonstrates the capability of the proposed sensor to detect arterial pulse under a preload. Arterial pulse refers to the sudden expansion of an artery due to the ejection of blood into the aorta. These results are not specific to any particular sex or gender without incorporating sex and gender into the study design. The sex and age of the subject are mentioned for the sake of completeness but are not relevant to the study.
Reporting on race, ethnicity, or other socially relevant groupings	Not relevant
Population characteristics	The 41 images of human hand with different gestures and/or arm sizes, which were used to train the vision-aided location prediction model in Supplementary Fig. S23, were collected from the volunteers (aged 20-50 years) of our study group. The arterial pulse data were captured from a healthy male, aged 25-30 years. Informed consent was obtained from the participants before conducting the experiments.
Recruitment	Volunteers are staff members of the study group.
Ethics oversight	The experiment of detecting arterial pulse was conducted with the approval of the Ethics Committee of Tongji Medical College, Huazhong University of Science and Technology. This Ethics Committee is constituted and functioned in accordance with ICH-GCP, GCP in China and Declaration of Helsinki (2013).

Note that full information on the approval of the study protocol must also be provided in the manuscript.

Field-specific reporting

Please select the one below that is the best fit for your research. If you are not sure, read the appropriate sections before making your selection.

[X] Life sciences [ ] Behavioural & social sciences [ ] Ecological, evolutionary & environmental sciences

For a reference copy of the document with all sections, see nature.com/documents/nr-reporting-summary-flat.pdf

Life sciences study design

All studies must disclose on these points even when the disclosure is negative.

Sample size	For the demonstration of arterial pulse detection as a proof-of-principle test, one healthy subject was selected.
Data exclusions	No data exclusion involved.
Replication	Arterial pulse detections were conducted more than three times and all testing results showed high similarity.
Randomization	The volunteer is a member of our study group, and there is no specific preparation for the human subject.
Blinding	For arterial pulse detection in Fig. 5, blinding the investigators was not required given that there was only one participant in the study. In Supplementary Fig. S23, the 41 human hand images, without any labels, were randomly allocated into the training and testing groups. The human subjects were briefed on the experimental process, but were not involved in data processing.

Reporting for specific materials, systems and methods

We require information from authors about some types of materials, experimental systems and methods used in many studies. Here, indicate whether each material, system or method listed is relevant to your study. If you are not sure if a list item applies to your research, read the appropriate section before selecting a response.

Materials & experimental systems

n/a	Involved in the study	
☒	☐	Antibodies
☒	☐	Eukaryotic cell lines
☒	☐	Palaeontology and archaeology
☒	☐	Animals and other organisms
☒	☐	Clinical data
☒	☐	Dual use research of concern
☒	☐	Plants

Methods

n/a	Involved in the study	
☒	☐	ChIP-seq
☒	☐	Flow cytometry
☒	☐	MRI-based neuroimaging

Plants

Seed stocks	This study does not involve plants.
Novel plant genotypes	This study does not involve plants.
Authentication	This study does not involve plants.
